# Differences in Characteristics and Ambulance Pathway Adherence Between Strokes and Mimics Presenting to a Large UK Centralized Hyper Acute Stroke Unit (HASU)

**DOI:** 10.3389/fneur.2021.646015

**Published:** 2021-05-10

**Authors:** Camilla Sammut-Powell, Christopher Ashton, Kyriaki Paroutoglou, Adrian Parry-Jones

**Affiliations:** ^1^Division of Informatics, Imaging and Data Science, Faculty of Biology, Medicine and Health, School of Health Sciences, University of Manchester, Manchester, United Kingdom; ^2^Salford Royal NHS Foundation Trust, Greater Manchester Integrated Stroke Delivery Network, Salford, United Kingdom; ^3^Salford Royal NHS Foundation Trust, Salford, United Kingdom; ^4^Geoffrey Jefferson Brain Research Centre, Manchester Academic Health Science Centre, Northern Care Alliance & University of Manchester, Manchester, United Kingdom; ^5^Division of Cardiovascular Science, Faculty of Biology, Medicine and Health, School of Medical Sciences, University of Manchester, Manchester, United Kingdom

**Keywords:** stroke recognition, stroke mimics, prehospital/EMS, pathway exclusions, suspected stroke

## Abstract

**Background:** In Greater Manchester (GM), prehospital clinicians use the Face Arm Speech Test (FAST) to identify suspected stroke patients alongside pathway exclusions. Within the centralized stroke service, patients with a suspected stroke are taken directly to a Hyper Acute Stroke Unit (HASU), often bypassing their local emergency department (ED). However, many of these patients are experiencing an illness that looks like a stroke but is not a stroke. The data collected in the prehospital setting is rarely used in research yet could give valuable insights into the performance of the pathway.

**Aim:** To evaluate the presenting symptoms and final diagnoses of prehospital suspected strokes and to evaluate the adherence of prehospital stroke pathway exclusions.

**Methods:** We analyzed data from all patients brought in by ambulance and admitted on the stroke pathway between 01/09/15 and 28/02/17. Patient demographics and all data recorded in the prehospital setting were evaluated to identify differences in stroke, TIA, and mimic patients. Pathway adherence was assessed according to whether the patient was local or out-of-area (OOA) and bypassed their local ED.

**Results:** A total of 4,216 suspected strokes were identified: 2,213 (52.5%) had a final diagnosis of stroke, 492 (11.7%) experienced a transient ischemic attack (TIA), and 1,511 (35.8%) were stroke mimics. There were 714 (16.9%) patients that were identified as having at least one pathway exclusion or were FAST negative, of which 270 (37.8%) experienced a stroke. The proportion of strokes was significantly lower in those with a pathway exclusion (41.8 vs. 53.5%; *p* < 0.001) and the proportion of breaches tended to be comparable or higher in the local population.

**Discussion:** There are high volumes of stroke mimics but identified differences indicate there is an opportunity to better utilize prehospital data. Ambulance clinicians were able to correctly overrule FAST negative results and the volume of these suggest that FAST alone may be too restrictive.

## Introduction

Identifying strokes can be challenging. The existence of stroke mimics and chameleons are well-documented: stroke mimics are non-strokes that present like strokes and chameleons are strokes that do not present with usual stroke-like symptoms (1). Brain imaging with computed tomography (CT) and/or magnetic resonance (MR) diffusion weighted imaging is considered the gold-standard for diagnosis (2) but MR is only available in hospital and although CT scanners can be installed in specially adapted ambulances, this approach is not widely available and more evidence is needed to prove cost-effectiveness (3, 4). In the vast majority of cases, prehospital decision making has focused on using simple stroke recognition tools with a high sensitivity and low specificity (5), leading to a high proportion of false positive cases, or “stroke mimics.”

**Figure 1:**
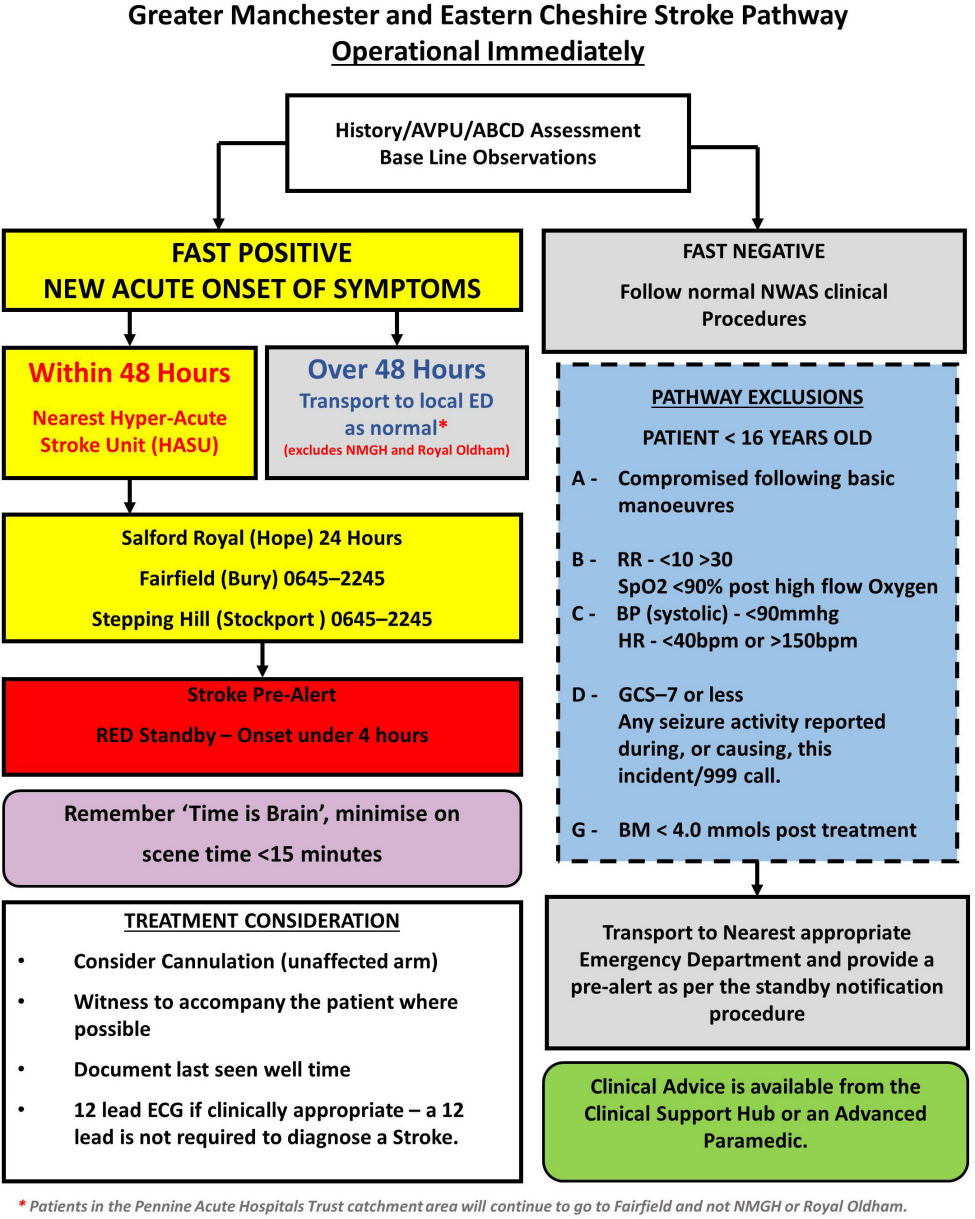
Stroke pathway followed by the North West Ambulance Service prehospital clinicians during the study period (1 September 2015–28 February 2017).

Stroke mimic rates have been reported to be between 4 and 43% in the prehospital setting (6), with common mimics being seizures, sepsis, and migraine (7–9). In UK ambulance services, the Face Arm Speech Test (FAST) is widely used for stroke recognition, however there are also service specific exclusions in the prehospital stroke pathway leading to inconsistencies across services (10). For example, the pathway for Greater Manchester (Figure 1) includes pathway exclusions intended to prioritize urgent treatment of unstable patients at the nearest Emergency Department (ED) over a potentially longer transfer to the nearest Hyper Acute Stroke Unit (HASU). For stroke, the maxim that “time is brain” emphasizes the importance of early identification and rapid transportation to a HASU, with immediate access to the full range of hyperacute stroke treatment. Yet, large volumes of mimics can result in delayed care, particularly in centralized services, and reduced resource availability for those that are experiencing a stroke (11, 12). Additionally, prehospital stroke care has been shown to affect in-hospital care processes, such as door-to-scan times (13, 14). Consequently, the prehospital setting has been identified as an area for improvement in stroke care (15).

A wealth of data is routinely collected in the prehospital setting but is often recorded on paper, making it difficult to collect for research purposes. A recent review identified only seven articles which discuss mimics in the prehospital setting (6), none of which report a full description of the prehospital data. A more recent study has reported prehospital data in much more detail, however, there was no interrogation to understand differences between those that bypassed their local emergency department and those local to the HASU (16), which is essential to understand prehospital decision making within a centralized service.

In this paper, we have collected and interrogated a large, combined prehospital and hospital dataset for sequential suspected stroke patients presenting by ambulance to a large HASU in an urban, centralized, UK stroke pathway. We describe the final diagnoses of these patients and compare the prehospital observations between strokes, TIAs, and stroke mimics. We also describe differences between patients local to the HASU and those that bypass their local emergency department for stroke care. Finally, we investigated the characteristics and outcomes of patients where prehospital clinicians incorrectly bypassed their local hospital to attend the HASU (pathway breaches) to further understand the reasons for these decisions and their impact.

## Methods

### Design and Data Sources

The study was a service evaluation using secondary, retrospective, observational data. A data sharing agreement was in place from 22/11/2017 between Salford Royal NHS Foundation Trust and the University of Manchester permitting analysis to be done in a secure data center at The University of Manchester as part of service improvement work. The study population consisted of patients that were brought in by ambulance (BIBA) to Salford Royal NHS Foundation Trust (SRFT) between 01 September 2015 and 28 February 2017 and had at least one of the following:

- Stroke team “significant event,” used as a record of stroke team review in the SRFT electronic patient record (EPR)
- “Stroke pathway” coded on arrival to ED
- “Cerebrovascular Accident/Transient Ischemic Accident” coded in final diagnosis
- Coded as a stroke admission in the EPR.

In order to maximize patient identification, we also cross-referenced the EPR to stroke medical on-call rotas, to identify all patients where a doctor on duty for stroke entered a clinical note in a patient's record. Patients that were transfers from another hospital arriving by ambulance were excluded. For each patient, a scanned copy of the ambulance patient report form (PRF) (recorded in the prehospital setting) was manually reviewed and entered into a database. The narrative information was examined for information on the presenting FAS symptoms, premorbid health and other neurological symptoms and translated into structured fields. Final diagnoses were identified from codes in the EPR. In cases where there was no clearly coded final diagnosis, the medical notes were reviewed by a consultant stroke physician (KP) to determine a final diagnosis.

The patients with the “FAST+” and/or “suspected stroke” box ticked “yes” on the PRF provided the final selection of the cohort of “suspected strokes.” Patients whose nearest ED was Salford Royal Hospital and had a final diagnosis of stroke but did not have “FAST+” nor “suspected stroke” box ticked “yes” on the PRF were considered the “missed strokes.” Patients whose nearest ED was Salford Royal Hospital and had a final diagnosis of stroke or TIA were defined as “All local strokes/TIAs (suspected or not).”

For the clinical observations recorded by the prehospital clinicians, there is space on the PRF for all observations to be recorded twice. The second set of observations can be recorded after administering treatment (e.g. oxygen, intravenous fluids) to record response, but in most cases only one observation is recorded. Therefore, we present only the first set of observations that are recorded. However, all breaches were evaluated using all observations taken. There was no field to identify if an airway was compromised, hence this information was not included in our dataset.

Patients were classified as “local” when Salford Royal Foundation Trust was also their nearest ED based on the postal code of their home address. Such patients would be conveyed to Salford Royal Foundation Trust regardless of whether they were brought on the stroke pathway and/or any exclusions applied. All other patients were classified as “out of area” and represent only those where they were brought to Salford Royal Foundation Trust instead of their local ED.

### Statistical Methods

Descriptive statistics of the baseline variables were evaluated within the suspected stroke population. Continuous variables are presented using the mean and standard deviation or median and interquartile range, as appropriate. Categorical variables are presented as counts and percentages. Differences in baseline characteristics between strokes, TIAs, and stroke mimics were assessed using ANOVA, Kruskal-Wallis tests, or chi-squared tests, as appropriate. Stroke mimics were dichotomized into neurological and non-neurological diagnoses.

Missing data within the recording of symptoms in the ambulance PRF was handled in the following manner: the symptoms were believed to be “truly missing” if none of the symptoms were indicated as present or absent. If at least one of the symptoms was recorded as present or absent, the remaining non-recorded symptom variables were filled in as “absent.” This was repeated for the past medical history variables. After this procedure was conducted, the proportion of missing data was calculated for each of the variables. A full list of variables and their descriptions can be found in Supplementary Material (Supplementary Table S1).

All analysis were performed using R v3.6.0 and RStudio v1.1.453.

## Results

A total of 5,365 patients were brought in by ambulance and admitted on the stroke pathway at Salford Royal Hospital between 1 September 2015 and 28 Feb 2017. Of these, 4,216 were deemed suspected strokes: 2,213 (52.5%) had a final diagnosis of stroke, 492 (11.7%) experienced a transient ischemic attack (TIA), and 1,511 (35.8%) were stroke mimics. A breakdown of the final diagnoses is shown in Table 1. Patients that experienced a stroke were on average 73.5 years old (SD 14.4) which was significantly older than stroke mimic patients whose mean age was 66.8 years old (SD 18.2; *p* < 0.001). Mimics such as migraine and functional disorders occurred in much younger populations and there were a higher proportion of females diagnosed with a stroke mimic compared to those experiencing a stroke (55.6 vs. 50.2%; *p* < 0.001). A higher proportion of patients had a stroke in the out-of-area population compared to those that were local (54.7 vs. 47.1%; *p* < 0.001) (Supplementary Table S2).

**Table 1:** Final diagnoses of the patients in the cohort, where *indicates a stroke mimic neurological diagnosis, with a summary of the sex and age of the patients.

**Final Diagnosis**	**Total**	**%**	**Female**	**%**	**Age**	**(SD)**
Stroke	2,213	52.5	1,112	50.2	73.5	14.4
TIA	492	11.7	259	52.6	70.9	15.1
Epilepsy*	244	5.8	118	48.4	68.7	14.4
Migraine*	241	5.7	163	67.6	46.5	15.3
Sepsis	218	5.2	116	53.2	75.9	14.5
Bell's palsy*	80	1.9	37	46.2	55.1	19.2
Syncope	79	1.9	48	60.8	73.4	15.2
Progressive symptoms	75	1.8	43	57.3	72.2	18.2
Limb/Facial pathology	69	1.6	38	55.1	67.5	18.1
Other medical pathology	68	1.6	40	58.8	66.5	17.1
Delirium*	67	1.6	43	64.2	77.1	14.3
Subarachnoid/Subdural/Epidural Hemorrhage*	65	1.5	35	53.8	75.2	13.6
Functional disorder	58	1.4	38	65.5	48.8	14.6
Hypotension/Hypoxia	56	1.3	33	58.9	75.8	16.9
Intracranial malignancy*	53	1.3	26	49.1	69.1	11.1
Cardiovascular event	43	1	21	48.8	72.1	15.7
Alcohol	25	0.6	<15	-	55.1	13
No Pathology Identified	18	0.4	<15	-	64.8	18.3
Hypoglycemia	17	0.4	<15	-	73.5	18.8
Allergy/Adverse reaction	17	0.4	<15	-	57.8	20.4
Anxiety disorder	<15	<0.4	<15	-	57.4	13.1
Meningoencephalitis/Mastoiditis	<15	<0.4	<15	-	57.2	25.8
All mimics (excl. TIA)	1,511	35.8	855	56.6	65.5	18.9

*Percentages of females were not presented for diagnoses where suppression was required for their count value*.

There were 4,055 (96.2%) patients who were recorded as FAST positive (FAST+ ticked “yes” or at least one FAS symptom recorded as positive in the narrative), 133 (3.2%) had FAST+ ticked “no” and had no FAS symptoms recorded as positive in the narrative and 28 (0.7%) had no record of FAS symptoms from either the FAST+ field or narrative. The FAST+ field was ticked “yes” in 3,666 (87.0%) patients and “no” in 398 (9.4%) patients but a positive FAS symptom was recorded in the narrative for 273 (Local: 146 vs. OOA:126, 1 missing location) of these, corresponding to an inconsistency in the FAST result in 6.5% of all suspected strokes. The proportion of these inconsistent recordings was much larger in local patients than OOA patients (14.1 vs. 4.2%; *p* < 0.001).

**Figure 2:**
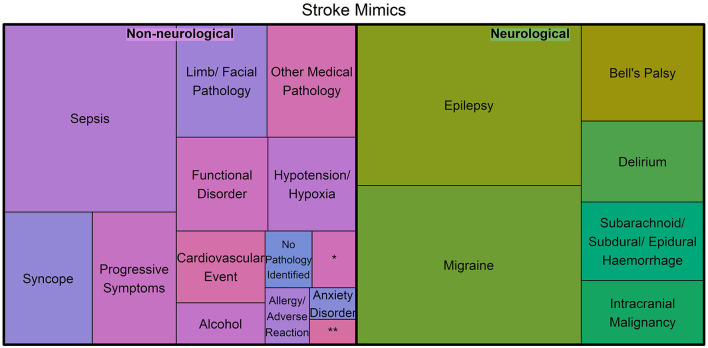
Treemap of the stroke mimic diagnoses categorized into neurological and non-neurological pathologies, where the area represents the proportion of mimics for each diagnosis (*Represents hypoglycemia and **Represents Meningoencephalitis/Mastoiditis).

A dichotomization into neurological and non-neurological diagnoses is given in Figure 2, where the area corresponds to the proportion. The proportion of neurological mimics (49.6%) was similar to that of the non-neurological mimics (50.4%). There was no significant difference in the proportion of neurological mimics between the OOA and the local patients (50.7 vs. 46.7%, respectively; *p* = 0.177).

The prehospital PRF data is presented in Table 2. Approximately 24% of ambulance PRFs did not contain any information on the patient's past medical history. Similarly, the symptoms were not recorded in 4% of the forms. The amount of missing data was consistent across the groups. There were a higher proportion of stroke mimics with a past medical history of epilepsy (8.3 vs. 2.7%, *p* < 0.001), migraine (4.7 vs. 1.2%, *p* < 0.001), alcohol misuse (2.7 vs. 1.5%, *p* = 0.023), and known mental health issues (10.0 vs. 7.3%, *p* = 0.007) compared with strokes. A higher proportion of stroke patients were recorded with atrial fibrillation (8.1 vs. 11.3%, *p* = 0.003), ischemic heart disease (14.9 vs. 19.2%, *p* = 0.001), and hypertension (32.0 vs. 44.1%, *p* < 0.001) compared with mimic patients. The proportion of patients with a previous stroke or TIA was higher in the stroke mimic group compared to the strokes (50.5 vs. 43.5%; *p* < 0.001).

Table 2Baseline past medical history, symptoms, and observations recorded on scene by prehospital clinicians for suspected stroke patients.

**Table:** 

	**Stroke**	**TIA**	**Stroke Mimic**	**All**	***p***
	***n* = 2,213**	***n* = 492**	***n* = 1,511**	***n* = 4,216**	
**Past medical history**	***n*** **= 1,682;**	***n*** **= 389;**	***n*** **= 1,135;**	***n*** **= 3,206;**	
	**Missing: 531 (24%)**	**Missing: 103 (20.9%)**	**Missing: 376 (24.9%)**	**Missing: 1,010 (24%)**	
Atrial fibrillation	**190 (11.3%)**	37 (9.5%)	87 (7.7%)	314 (9.8%)	**0.006**
Ischemic heart disease	**323 (19.2%)**	70 (18.0%)	157 (13.8%)	550 (17.2%)	**0.001**
Hypertension	**781 (44.1%)**	144 (37.0%)	344 (30.3%)	1,229 (38.3%)	**<0.001**
Diabetes	379 (22.5%)	99 (25.4%)	248 (21.9%)	726 (22.6%)	0.338
Epilepsy	46 (2.7%)	12 (3.1%)	**114 (10.0%)**	172 (5.4%)	**<0.001**
TIA or stroke	732 (43.5%)	**206 (53.0%)**	564 (49.7%)	1502 (46.8%)	**<0.001**
Migraine	21 (1.2%)	8 (2.1%)	**63 (5.6%)**	92 (2.9%)	**<0.001**
Dementia	218 (13.0%)	47 (12.1%)	154 (13.6%)	419 (13.1%)	0.741
Mental health	123 (7.3%)	25 (6.4%)	**128 (11.3%)**	276 (8.6%)	**<0.001**
Substance misuse	11 (0.7%)	<5 (<1.3%)	8 (0.7%)	<24 (<0.8%)	0.922
Alcohol misuse	25 (1.5%)	5 (1.3%)	**36 (3.2%)**	66 (2.1%)	**0.004**
**Symptom*****s***	***n*** **= 2,121;**	***n*** **= 472;**	***n*** **= 1,454;**	***n*** **= 4,047;**	
	**Missing: 92 (4.2%)**	**Missing: 20 (4.0%)**	**Missing: 57 (3.8%)**	**Missing: 169 (4.0%)**	
Seizure	26 (1.2%)	<5 (<1.1%)	**99 (6.8%)**	<130 (<3.3%)	**<0.001**
Unilateral leg weakness	**1,046 (49.3%)**	140 (29.7%)	489 (33.6%)	1,675 (41.4%)	**<0.001**
Reduced mobility	**543 (25.6%)**	82 (17.4%)	295 (20.3%)	920 (22.7%)	**<0.001**
Unsteadiness	**301 (14.2%)**	53 (11.2%)	156 (10.7%)	510 (12.6%)	**0.006**
Visual disturbance/changes	143 (6.7%)	**55 (11.7%)**	135 (9.3%)	333 (8.2%)	**<0.001**
Gaze deviation	**97 (4.6%)**	5 (1.1%)	31 (2.1%)	133 (3.3%)	**<0.001**
Vomiting	172 (8.1%)	19 (4.0%)	**144 (9.9%)**	335 (8.3%)	**<0.001**
Difficulty swallowing	57 (2.7%)	8 (1.7%)	39 (2.7%)	104 (2.6%)	0.442
Dizzy	193 (9.1%)	54 (11.4%)	147 (10.1%)	394 (9.7%)	0.250
Leaning to one side	206 (9.7%)	33 (7.0%)	**164 (11.3%)**	403 (10.0%)	**0.022**
Fall	**486 (22.9%)**	44 (9.3%)	228 (15.7%)	758 (18.7%)	**<0.001**
Headache	418 (19.7%)	123 (26.1%)	**438 (30.1%)**	979 (24.2%)	**<0.001**
Loss of consciousness	96 (4.5%)	9 (1.9%)	**151 (10.4%)**	256 (6.3%)	**<0.001**
Generalized weakness	34 (1.6%)	12 (2.5%)	**61 (4.2%)**	107 (2.6%)	**<0.001**
Memory loss	46 (2.2%)	15 (3.2%)	22 (1.5%)	83 (2.1%)	0.073
Behavioral changes	80 (3.8%)	20 (4.2%)	**89 (6.1%)**	189 (4.7%)	**0.004**
Confusion	350 (16.5%)	93 (19.7%)	**288 (19.8%)**	731 (18.1%)	**0.025**
Loss or change in sensation	312 (14.7%)	**99 (21.0%)**	285 (19.6%)	696 (17.2%)	**<0.001**

**Table:** 

**First Observations**	**Value**	**% Missing**	**Value**	**% Missing**	**Value**	**% Missing**	**Value**	**% Missing**	
Blood glucose (mmol/L)	**7.8 (2.9)**	6.5	7.3 (2.5)	5.5	7.5 (2.8)	6.6	7.7 (2.8)	6.4	**<0.001**
Systolic blood pressure	**156.9 (32.3)**	5.4	155.1 (31.8)	5.7	148.0 (30.0)	6.3	153.5 (31.7)	5.7	**<0.001**
Diastolic blood pressure	**85.7 (18.4)**	7.2	85.5 (18.4)	5.1	83.0 (17.9)	5.9	84.7 (18.4)	6.5	**<0.001**
Temperature (°C)	36.5 (0.6)	6.0	36.5 (0.5)	4.1	**36.7 (0.8)**	5.4	36.6 (0.7)	5.6	**<0.001**
GCSE^†^	4 (4–4)	1.8	4 (4–4)	1.2	4 (4–4)	0.8	4 (4–4)	1.4	**<0.001**
GCSV^†^	5 (4–5)	1.9	5 (5–5)	1.2	5 (4–5)	0.9	5 (4–5)	1.5	**<0.001**
GCSM^†^	6 (6–6)	1.7	6 (6–6)	1.2	6 (6–6)	0.9	6 (6–6)	1.4	**<0.001**
Total GCS^†^	15 (14–15)	2.0	15 (15–15)	1.2	15 (14–15)	1.1	15 (14–15)	1.6	**<0.001**
Heart rate	83.0 (19.7)	6.6	81.4 (17.6)	4.9	**84.8 (19.5)**	6.1	83.4 (19.4)	6.2	**0.001**
Respiratory rate	17.9 (4.3)	3.4	17.4 (3.4)	2.6	**18.1 (4.3)**	4.2	17.9 (4.2)	3.6	**0.007**
LOC^†^	1 (1–1)	2.5	1 (1–1)	1.6	1 (1–1)	2.3	1 (1–1)	2.3	**<0.001**
Pain^†^	0 (0–0)	21.8	0 (0–0)	16.1	0 (0–1)	21.9	0 (0–0)	21.2	**<0.001**
SPO2 air	96.3 (3.6)	4.3	**96.8 (2.4)**	3.3	96.3 (3.4)	4.9	96.3 (3.4)	4.4	**0.007**
SPO2 oxygen	93.6 (15.0)	95.1	97.3 (1.9)	96.7	91.0 (22.5)	93.2	92.7 (18.4)	94.6	0.356
Pupil equal	1,917 (93.2%)	7.0	**453 (97.4%)**	5.5	1,333 (94.7%)	6.9	3,703 (94.2%)	6.8	**0.001**
Cap Refill	46 (2.2%)	4.5	5 (2.2%)	3.3	40 (1.1%)	4.9	91 (2.3%)	4.5	0.082

*The group with the largest proportion or measure is indicated in bold when the groups were found to be statistically significantly different*.

*First observations were presented as the mean (standard deviation) or count (%) unless indicated by*.

† *symbol, indicating the median (lower quartile - upper quartile) is presented. GCS, Glasgow coma scale; LOC: loss of consciousness on scale 1 = Alert, 2 = Voice, 3 = Pain, 4 = Unresponsive; Pain: pain on scale 0–10. SPO2 air, saturation of peripheral oxygen on air; SPO2 oxygen: saturation of peripheral oxygen after administration of oxygen*.

Stroke mimics had lower blood sugar levels, lower blood pressure (systolic and diastolic), higher temperature, higher heart rate, more pain, and tended to have equal pupil reaction. The distribution of the subcategories of GCS was different across the groups which in turn contributed to a difference in the overall GCS as demonstrated in Supplementary Figures S1, S2. Similarly, the distribution of pain was different across the groups, with a larger proportion reporting higher pain scores in the mimics group (Supplementary Figure S3).

**Figure 3:**
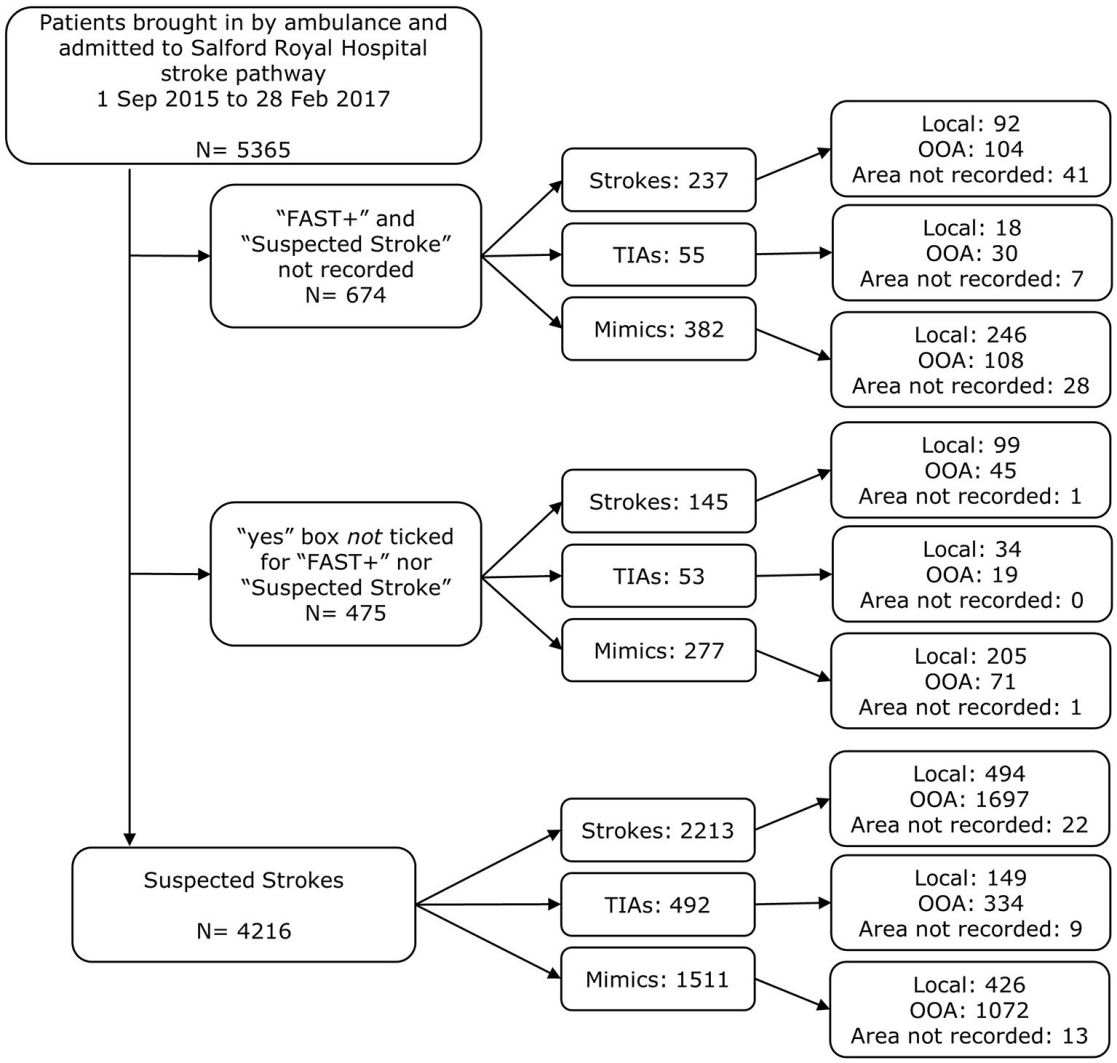
Exclusion diagram for identifying patients as prehospital suspected strokes brought in by ambulance on the stroke pathway, with details of whether the patient was out-of-area or local to Salford Royal Foundation Trust. Indications of stroke were taken to be “FAST+” and/or “suspected stroke” ticked “yes” in the ambulance PRF.

From the 5,365 patients initially identified (suspected and non-suspected), 685 had a final diagnosis of stroke and were local to the HASU. Of these, 494 were considered “suspected strokes” (Figure 3), corresponding to a sensitivity of 72.1%. From the “missed strokes” (i.e., those that were not suspected strokes but had a final diagnosis of stroke and were local to the HASU) with an onset-to-arrival time available (7/191), fewer than five arrived at hospital within 4 h from onset.

There were 714 (16.9%) suspected strokes that were identified as having at least one pathway exclusion or were FAST negative, of which 270 (37.8%) experienced a stroke and 130 (18.2%) experienced a TIA. The proportion of strokes was significantly lower in those with a pathway exclusion (excl:41.8% vs. no excl:53.5%; *p* < 0.001) and the proportion of mimics that were neurological was significantly higher in those that had an exclusion compared to those without (61.7 vs. 48.7%, respectively; *p* = 0.002). Within the local population, there was no significant difference in these proportions (59.3 vs. 45.2%; *p* = 0.08). Overall, the proportions of strokes/TIAs and mimics for each exclusion were small (Table 3). The criterion most breached was seizure activity, with 99 (6.8%) of the mimic patients having experienced a seizure which was significantly higher than those that experienced a stroke/TIA (6.8 vs. 1.1%; *p* < 0.001). Patients younger than 16 or with a respiratory rate below 10 were very infrequent and are therefore not reported. The proportion of breaches tended to be comparable or higher in the local population compared to the OOA population. In both the local and OOA subgroups, the proportion of mimic patients with a breach was approximately double of that in the stroke and TIA group. Notably, the seizure activity breach rates were similar between OOA and local patients.

**Table 3:** Percentages of pathway exclusion breaches and FAST negative patients by final diagnosis and area.

**Pathway Breach**	**OOA Suspected Strokes**			**Local Suspected Strokes**			**All local strokes/TIAs (suspected or not)**
	**Stroke/TIA**	**Mimics**	**Total**	**Stroke/TIA**	**Mimics**	**Total**	**Total**
	**(*n* = 2,031)**	**(*n* = 1,072)**	**(*n* = 3,103)**	**(*n* = 643)**	**(*n* = 426)**	**(*n* = 1,069)**	**(*n* = 886)**
Respiratory rate >30	0.9%	1.0%	1.0%	1.9%	2.4%	2.1%	1.8%
	Missing:29	Missing:19	Missing:48	Missing:12	Missing:13	Missing:25	Missing:19
Systolic blood pressure <90	0.9%	0.8%	0.8%	0.8%	<1.0%	0.8%	0.9%
	Missing:44	Missing:31	Missing:75	Missing:29	Missing:20	Missing:49	Missing:36
SPO2 <90	1.0%	1.1%	1.1%	1.1%	1.2%	1.1%	1.2%
	Missing:28	Missing:19	Missing:47	Missing:14	Missing:6	Missing:20	Missing:22
Seizure activity	1.1%	6.6%	3.0%	1.0%	6.7%	3.2%	1.4%
	Missing:84	Missing:34	Missing:118	Missing:25	Missing:22	Missing:47	Missing:40
Heart rate <40	0.4%	0.5%	0.4%	1.1%	<1.0%	1.0%	0.8%
	Missing:54	Missing:27	Missing:81	Missing:18	Missing:17	Missing:35	Missing:31
Heart rate >150	0.4%	0.4%	0.4%	<0.8%	<1.0%	0.7%	0.6%
	Missing:54	Missing:27	Missing:81	Missing:18	Missing:17	Missing:35	Missing:31
GCS <8	0.6%	0.8%	0.7%	0.9%	2.4%	1.5%	1.9%
	Missing:22	Missing:4	Missing:26	Missing:6	Missing:5	Missing:11	Missing:12
Blood glucose <4.0 mmol	0.5%	1.0%	0.6%	<0.8%	<1.0%	0.6%	0.6%
	Missing:100	Missing:52	Missing:152	Missing:25	Missing:22	Missing:47	Missing:45
At least one exclusion	6.2%	11.5%	7.5%	7.9%	12.7%	9.2%	8.9%
	Missing:262	Missing:134	Missing:396	Missing:86	Missing:67	Missing:153	Missing:132
FAST negative	6.3%	6.6%	6.4%	19.7%	19.5%	19.6%	28.9%
	Missing:63	Missing:39	Missing:102	Missing:28	Missing:21	Missing:49	Missing:191
FAST negative with no FAS symptoms recorded	2.0%	2.5%	2.2%	4.1%	7.2%	5.3%	10.4%
	Missing:63	Missing:39	Missing:102	Missing:28	Missing:21	Missing:49	Missing: 191
At least one exclusion or FAST negative	13.0%	20.4%	15.5%	29.2%	34.7%	31.4%	39.9%
	Missing:272	Missing:156	Missing:428	Missing:85	Missing:69	Missing:154	Missing:229
At least one exclusion or FAST negative (with no FAS symptoms recorded)	8.5%	16.3%	11.2%	12.5%	22.5%	16.5%	21.4%
	Missing:284	Missing:160	Missing:444	Missing:101	Missing:75	Missing:176	Missing:250

A total of 2,302 patients had a recorded onset-to-arrival time, of which 1,689 (73.4%) arrived within 4 h. The proportion of patients that arrived within 4 h was not significantly different between OOA and local patients (73.3 vs. 73.7%; *p* = 0.871). Within the OOA patients, having an exclusion (not including FAST negative) was associated with higher proportion of patients arriving within 4 h from onset (no excl: 73.0 vs. excl: 83.3%; *p* = 0.016). Out of the 217 OOA breaches with onset-to-arrival, 82 (37.8%) were strokes arriving within 4 h.

## Discussion

This is a large study describing stroke mimic rates in the prehospital setting within a centralized service, detailing differences in rates according to locality to the HASU and is the first study which reports pathway breaches. As well as supporting findings in the literature, e.g., the prehospital blood pressure being higher in the stroke population (17), our results contribute novel understanding around baseline variables and pathway adherence, not previously investigated.

Overall, we have shown several differences between the stroke, TIA, and mimic populations. The mimics consisted equally of neurological and non-neurological pathologies and tended to be younger with a higher proportion of females. A higher proportion of stroke patients had a cardiovascular medical history (excluding stroke/TIA) and mimics patients were more likely to have a history of mimics (e.g., epilepsy, migraine, alcohol misuse). From the first set of clinical observations, the blood glucose and blood pressure were higher in the stroke patients. Finally, seizure activity appeared to be the most breached exclusion which was significantly higher in the mimic patients.

While we cannot interpret causal relationships, we can speculate that the higher proportion of patients with a previous history of a TIA or stroke in the mimic population could be indicative of the decision-making. It could influence the initial decision to call emergency services in the community and the decisions made by prehospital clinicians; it is well known that patients who have already experienced a stroke or TIA have a higher risk of a recurrent stroke (18), hence may motivate the decision to direct the patient to the HASU.

Although pathways are modified to improve efficiency and safety, breaches occur. In some cases, these turn out to be stroke chameleons. It is useful to understand what elements of the pathway are breached when arriving at both HASUs and District Stroke Centers, and how many of these are strokes. However, since practice across ambulance services is heterogeneous, new practices are not well disseminated and paramedics feel they can benefit from both more training and feedback on prehospital stroke care (19).

The proportions of patients with pathway breaches were similar for strokes and mimics, reflective of their intended purpose of ensuring prompt arrival at the nearest ED to treat unstable patients, rather than to aid diagnosis. The lower rates in the OOA patients indicate that the exclusion criteria are usually followed, reducing the number of OOA patients arriving at the HASU with an exclusion. However, identification of our cohort at the HASU means we are unable to measure how often a prehospital clinician is applying the pathway exclusions and choosing to not put the patient on the stroke pathway because of this.

Breaches may occur because it is considered that a rare presentation of stroke is more likely than a common presentation of a rarer condition (1). Ambulance clinicians do not routinely receive feedback unless there is investigation into a serious clinical issue or complaint. However, the perception of risk within a risk averse ambulance service/organization has been reported to influence prehospital decision making toward “erring on the side of caution” (20). This could be inferred from the larger proportion of mimics presented to the stroke pathway when they are local since they would have been directed to Salford Royal Foundation Trust regardless. However, the breach rates for seizures were approximately equal across the local and OOA populations. This could be due to one of the following: (1) seizures tend to be directed to the head injury unit and the only specialist unit in GM is at Salford Royal Foundation Trust; (2) the seizures resolve on the way to the hospital; (3) ambulance clinicians act “risk averse” when faced with seizures and think the best place is Salford Royal Foundation Trust.

The large proportion of FAST negative strokes and TIAs observed in the local population is a concern for UK ambulance services. The difference in the rates between OOA and local patients could suggest that there are a number of FAST negative strokes and TIAs which will be transported to their local hospital rather than the HASU. Although the mortality within the local FAST negative patients appears low (Supplementary Table S2), we could expect worse outcomes for patients whose local hospital is not a HASU. Notably, however, 6.3% of the OOA strokes were correctly diverted to the HASU, with the prehospital clinician overruling the FAST result as being a pathway exclusion. This should be commended and the crews' decisions around these FAST negative presentations should be investigated to consider how the pathway could be adapted. It could also inform additional training for paramedics, which they would welcome (19).

It could be argued that the local population with a final diagnosis of stroke/TIA acts as a control group for the breach criteria in stroke/TIA patients. Doing so suggests that there are a number of FAST negative strokes/TIAs that were not diverted to the HASU, indicating that using FAST alone may be too restrictive as a stroke recognition tool. This adds to the evidence that FAST leads to too many false negatives (21, 22) and causes the patient to be directed to the wrong point of care. On the other hand, rates were comparable across the exclusion criteria suggesting that the ambulance clinicians are often able to correctly divert strokes with exclusions. However, without knowledge of the OOA patients that weren't diverted, we cannot make any concrete conclusions.

The percentage of strokes was much lower in Salford Royal Hospital than previously reported in centralized services (6, 23). Further, the rates of mimics were often considerably higher, for example, epilepsy was found to be the primary diagnoses for 32.6% of the mimic population, which is over ten times larger than reported in McClelland et al. (6). This could in part be due to the different approaches in verifying the final diagnoses, however, it remains a clear difference between the groups and an indication that there needs to be a thorough evaluation of UK services.

Discriminating well between strokes and mimics is particularly important in centralized stroke services. Our results demonstrate that there is a missed opportunity to utilize the routinely collected data to evaluate pathways and potentially improve stroke recognition at no additional burden.

## Limitations

This was a retrospectively collected cohort and we decided to exclude patients that had no positive indication of stroke (“yes” box not ticked for “FAST+” nor “Suspected stroke”) from the “suspected stroke” population. However, this may have excluded some patients that were brought to Salford Royal Hospital on the stroke pathway, with a primary impression of stroke. In addition, to fully understand adherence to the prehospital stroke pathway, it is necessary to have the data for patients that were first considered a possible stroke at point of triage during the 999 call and then ruled out from using the pathway. This “negative” population is not possible to obtain retrospectively since this level of detail is not routinely recorded.

The data are taken from a single site and consequently may not be fully generalizable to the stroke population, particularly where there is not a centralized stroke service and/or differences to the ambulance pathway. Further, recording in the prehospital setting is not always consistent between scenarios. In some cases, the data may be recorded on scene and other occasions, it may be on the way to the hospital. It may be that a patient presented with symptoms on scene, but these resolve on the way to the hospital in which case the reporting may or may not reflect this. Further, the data was primarily recorded to inform the patient's care. Consequently, fields which have been evaluated for this study may not have been considered relevant by the prehospital clinician or recording may not have been prioritized.

Finally, stroke pathways can differ across centers which can influence the categorization of final diagnosis. For example, although subarachnoid hemorrhage is considered a subtype of stroke, it is managed via an entirely different pathway, led by the Neurosurgical Department within Salford Royal. Consequently, for the purposes of reviewing our stroke pathway, we have considered subarachnoid hemorrhage separately.

## Data Availability Statement

The datasets presented in this article are not readily available because Pseudonymised patient-level data was used. Access to the data would need to be approved by the data provider. Requests to access the datasets should be directed to adrian.parry-jones@srft.nhs.uk.

## Ethics Statement

Ethical review and approval was not required for the study on human participants in accordance with the local legislation and institutional requirements. Written informed consent for participation was not required for this study in accordance with the national legislation and the institutional requirements.

## Author Contributions

CA and AP-J conceived the ideas for the study. KP assisted in the data collection and review of the data. CS-P performed the analysis. The manuscript was written by CS-P and edited by CA and AP-J. All authors reviewed the final version of the manuscript.

## Conflict of Interest

The authors declare that the research was conducted in the absence of any commercial or financial relationships that could be construed as a potential conflict of interest.

## References

[1] Fernandes PM Whiteley WN Hart SR Al-Shahi Salman R. Strokes: mimics and chameleons. Pract Neurol. (2013) 13:21–8. 10.1136/practneurol-2012-000465 23315456

[2] Vymazal J Rulseh AM Keller J Janouskova L. Comparison of CT and MR imaging in ischemic stroke. Insights Imaging. (2012) 3:619–27. 10.1007/s13244-012-0185-9 23055115 PMC3505566

[3] Ehntholt MS Parasram M Mir SA Lerario MP. Mobile stroke units: bringing treatment to the patient. Curr Treat Options Neurol. (2020) 22:5. 10.1007/s11940-020-0611-0 32025945

[4] Harris J. A review of mobile stroke units. J Neurol. (2020) 10.1007/s00415-020-09910-4. [Epub ahead of print]. 32424611

[5] Zhelev Z Walker GB Henschke N Fridhandler J Yip S. Prehospital stroke scales as screening tools for early identification of stroke and transient ischemic attack. Stroke. (2019) 50:E285–6. 10.1161/STROKEAHA.119.026527 30964558 PMC6455894

[6] McClelland G Rodgers H Flynn D Price CI. The frequency, characteristics and etiology of stroke mimic presentations: a narrative review. Eur J Emerg Med. (2019) 26:2–8. 10.1097/MEJ.0000000000000550 29727304

[7] Kim SJ Kim DW Kim HY Roh HG Park JJ. Seizure in code stroke: stroke mimic and initial manifestation of stroke. Am J Emerg Med. (2019) 37:1871–75. 10.1016/j.ajem.2018.12.051 30598373

[8] Gibson LM Whiteley W. The differential diagnosis of suspected stroke. J R Coll Physicians Edinb. (2013) 43:114–8. 10.4997/JRCPE.2013.205 23734351

[9] Southerland AM. Clinical evaluation of the patient with acute stroke. Contin Lifelong Learn Neurol. (2017) 23:40–61. 10.1212/CON.0000000000000437 28157743

[10] McClelland G Rogers H Price CI. A survey of pre-hospital stroke pathways used by UK ambulance services. Int J Stroke. (2018) 13(3 Suppl):35. 10.1177/174749301880110827145795

[11] Ifergan H Amelot A Ismail M Gaudron M Cottier JP Narata AP. Stroke-mimics in stroke-units. Evaluation after changes imposed by randomized trials. Arq Neuropsiquiatr. (2020) 78:88–95. 10.1590/0004-282x20190154 32159722

[12] Dawson A Cloud GC Pereira AC Moynihan BJ. Stroke mimic diagnoses presenting to a hyperacute Stroke unit. Clin Med J R Coll Physicians London. (2016) 16:423–6. 10.7861/clinmedicine.16-5-423 27697802 PMC6297311

[13] Ashton C Sammut-Powell C Birleson E Mayoh D Sperrin M Parry-Jones AR. Implementation of a prealert to improve in-hospital treatment of anticoagulant-associated strokes: analysis of a prehospital pathway change in a large UK centralised acute stroke system. BMJ Open Qual. (2020) 9:1–3. 10.1136/bmjoq-2019-000883 32423973 PMC7239536

[14] Sheppard JP Mellor RM Greenfield S Mant J Quinn T Sandler D. The association between prehospital care and in-hospital treatment decisions in acute stroke: A cohort study. Emerg Med J. (2015) 32:93–99. 10.1136/emermed-2013-203026 24099829 PMC4316848

[15] Fassbender K Walter S Grunwald IQ Merzou F Mathur S Lesmeister M. Prehospital stroke management in the thrombectomy era. Lancet Neurol. (2020) 19:601–610. 10.1016/S1474-4422(20)30102-2 32562685

[16] McClelland G Flynn D Rodgers H Price C. Positive predictive value of stroke identification by ambulance clinicians in North East England: a service evaluation. Emerg Med J. (2020) 37:474–9. 10.1136/emermed-2019-208902 32385043

[17] Gioia LC Zewude RT Kate MP Liss K Rowe BH Buck B. Prehospital systolic blood pressure is higher in acute stroke compared with stroke mimics. Neurology. (2016) 86:2146–53. 10.1212/WNL.0000000000002747 27194383 PMC4898317

[18] Coull AJ Lovett JK Rothwell PM. Population based study of early risk of stroke after transient ischaemic attack or minor stroke: implications for public education and organisation of services. Br Med J. (2004) 328:326–8. 10.1136/bmj.37991.635266.44 14744823 PMC338101

[19] McClelland G Flynn D Rodgers H Price C. A survey of UK paramedics' views about their stroke training, current practice and the identification of stroke mimics. Br Paramed J. (2017) 2:4–15. 10.29045/14784726.2017.2.1.4

[20] Brunton L Boaden R Knowles S Ashton C Parry-Jones AR. Pre-hospital stroke recognition in a UK centralised stroke system: a qualitative evaluation of current practice. Br Paramed J. (2019) 4:31–9. 10.29045/14784726.2019.06.4.1.31 33328826 PMC7706776

[21] Gulli G Markus HS. The use of FAST and ABCD2 scores in posterior circulation, compared with anterior circulation, stroke and transient ischemic attack. J Neurol Neurosurg Psychiatry. (2012) 83:228–9. 10.1136/jnnp.2010.222091 21205982

[22] Zhao H Pesavento L Coote S Rodrigues E Salvaris P Smith K. Ambulance clinical triage for acute stroke treatment: paramedic triage algorithm for large vessel occlusion. Stroke. (2018) 49:945–51. 10.1161/STROKEAHA.117.019307 29540611

[23] Neves Briard J Zewude RT Kate MP Rowe BH Buck B Butcher K. Stroke mimics transported by emergency medical services to a comprehensive stroke center: the magnitude of the problem. J Stroke Cerebrovasc Dis. (2018) 27:2738–45. 10.1016/j.jstrokecerebrovasdis.2018.05.046 30056002

