# Supplementary material for: Differences in Characteristics and Ambulance Pathway Adherence Between Strokes and Mimics Presenting to a Large UK Centralized Hyper Acute Stroke Unit (HASU)
Source: Front Neurol. 2021 May 10;12:646015. doi: 10.3389/fneur.2021.646015 (PMC8143189; doi:10.3389/fneur.2021.646015)
Supplement: Supplementary file 1 [file Data_Sheet_1.docx]

Supplementary Material

# Supplementary Figures and Tables

## Supplementary Figures


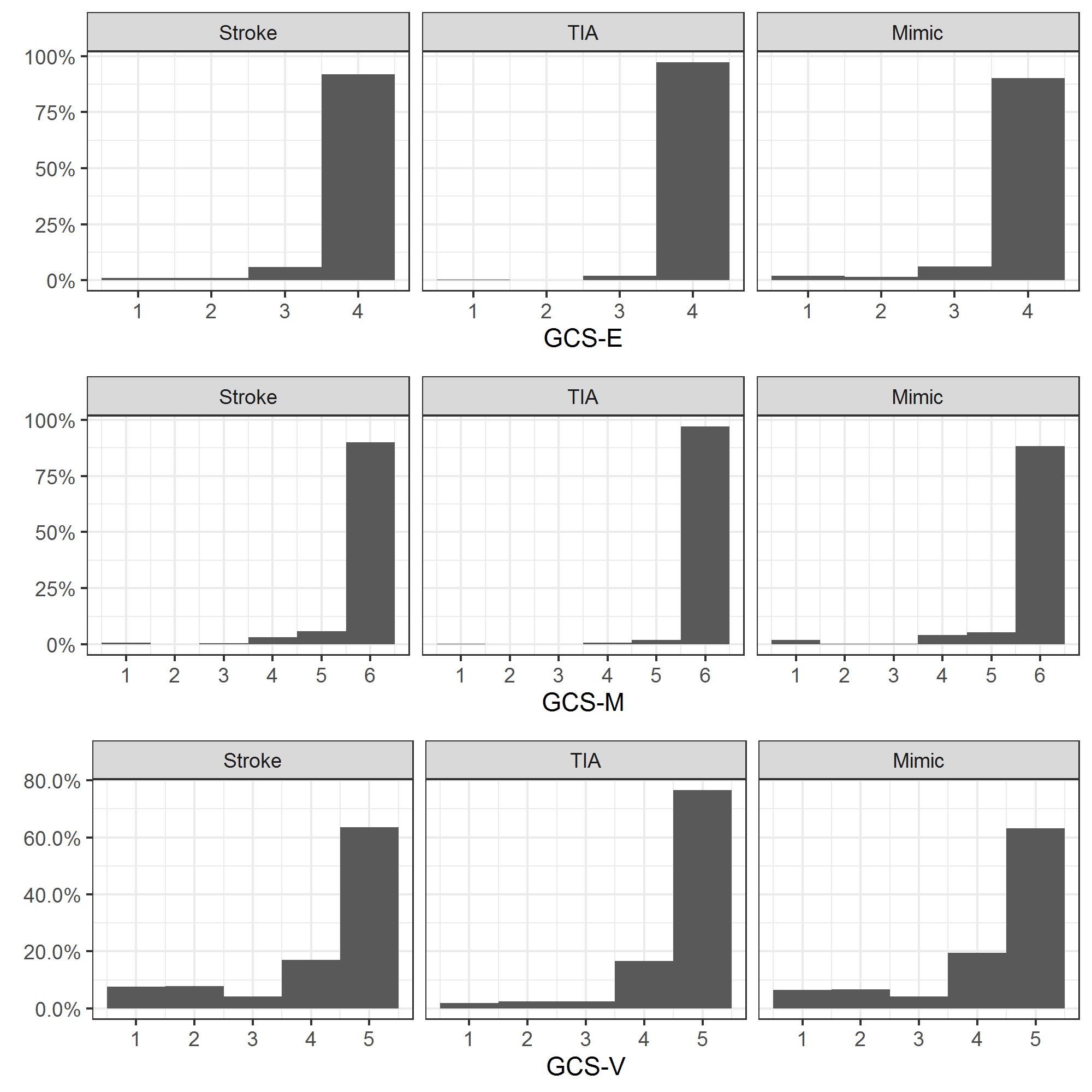


**Supplementary Figure S1.** Distribution of elements of GCS recorded prehospital for suspected strokes, categorized by final diagnosis of stroke, TIA and mimics.


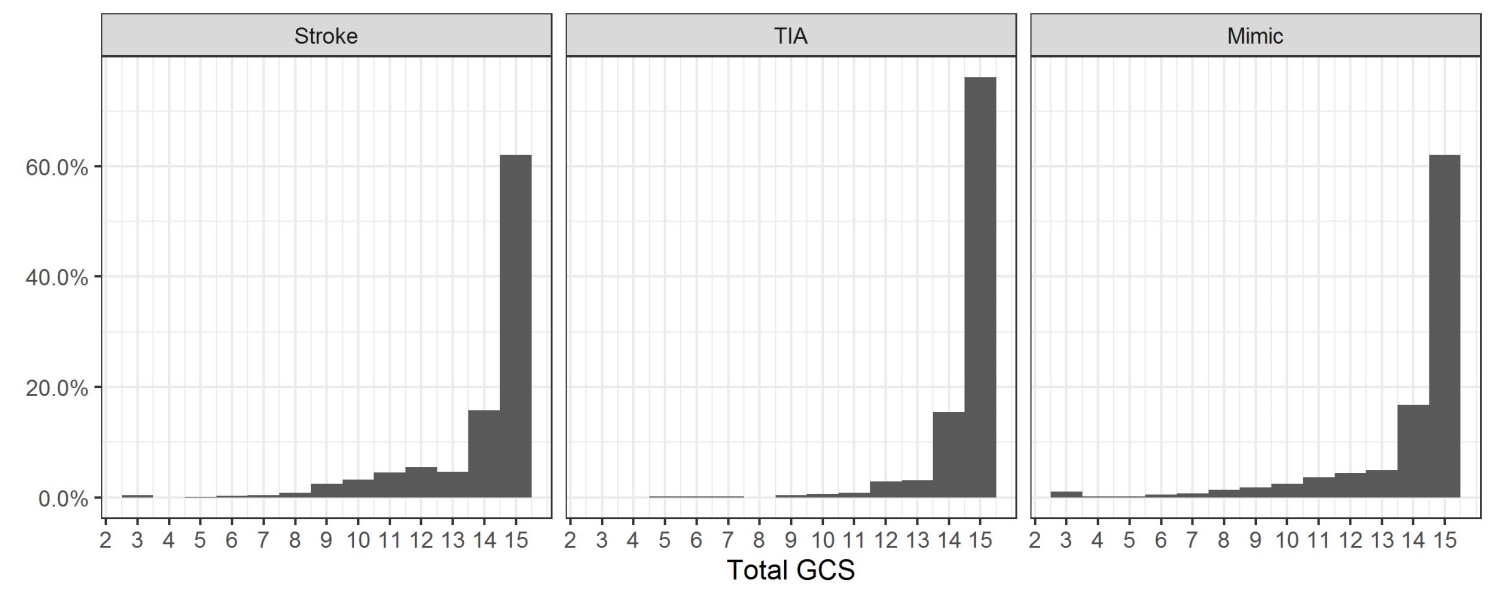


**Supplementary Figure S2.** Distribution of total GCS recorded prehospital for suspected strokes, categorized by final diagnosis of stroke, TIA and mimics.


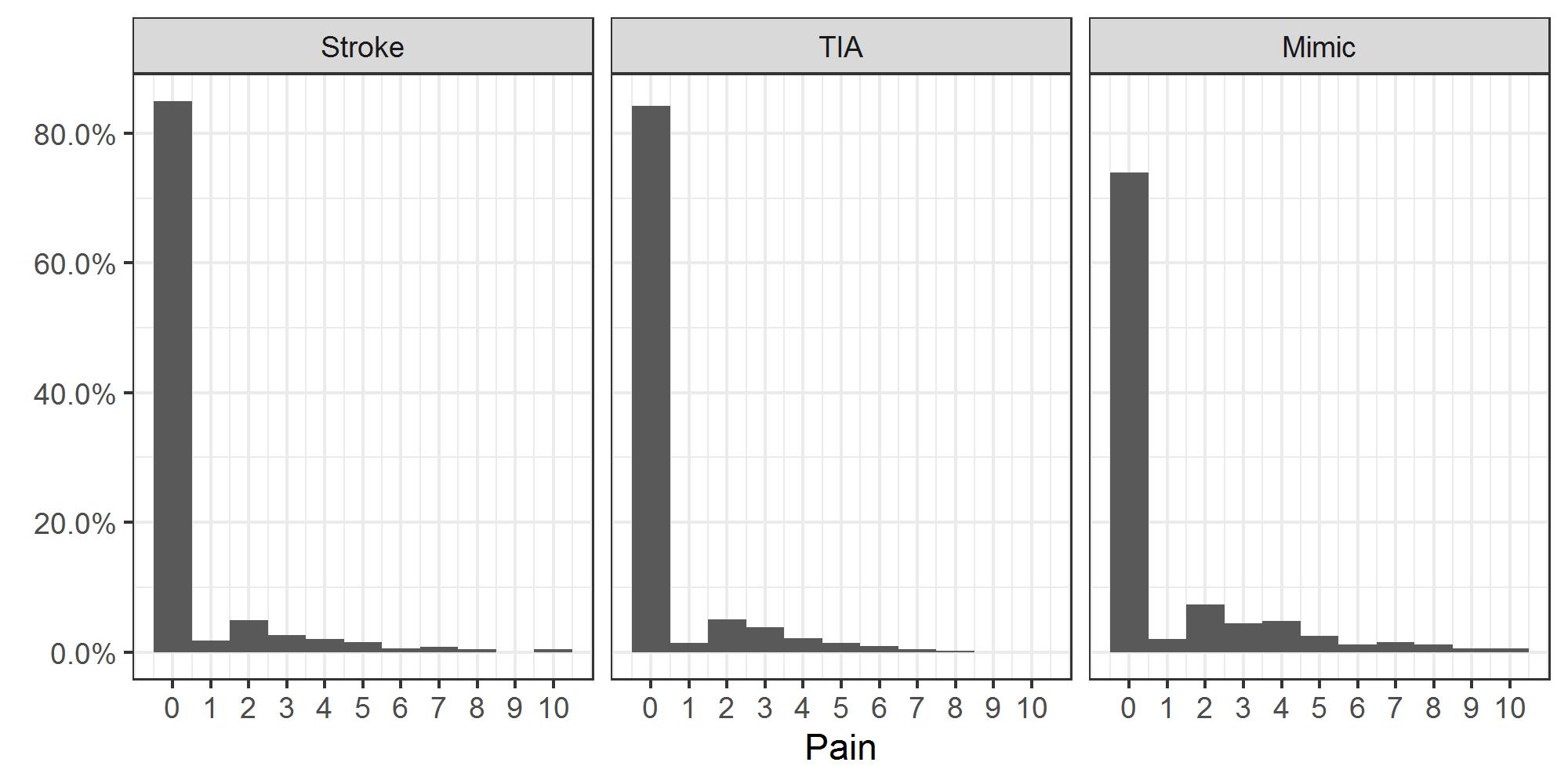


**Supplementary Figure S3.** Distribution of pain score recorded prehospital for suspected strokes, categorized by final diagnosis of stroke, TIA and mimics.

## Supplementary Tables

**Supplementary Table S1**: Full description of variables recorded in the patient record form

| **Category** | **Field** | **Notes** |
| --- | --- | --- |
| Previous History | PMH ischemic heart disease | Include angina, previous MI |
|  | PMH atrial fibrillation or flutter | Prior history of atrial fibrillation, atrial flutter. Includes permanent and paroxysmal |
|  | PMH hypertension | Documented as high blood pressure or hypertension |
|  | PMH diabetes | Documented as type 1 or 2 diabetes mellitus |
|  | PMH Epilepsy | Prior history of epilepsy. Include any patient with a prior history of seizures. Do not include if incident is first ever seizure. |
|  | PMH Stroke or TIA | Prior history of stroke or TIA, do not include current incident |
|  | PMH Migraine | Prior history of migraine, with or without aura. Do not include if only says ‘headaches’ |
|  | PMH Dementia | Prior history of any type of dementia |
|  | PMH Mental health | Prior history of any mental health condition eg: depression, anxiety, psychosis, schizophrenia but NOT dementia |
|  | Substance misuse | Mark yes if any prior or incident related drug abuse |
|  | Alcohol misuse | History of any prior or incident related excessive or problematic alcohol use |
| Other symptoms:  as part of presenting incident (include things that patient reports but have resolved or not objectively seen by the paramedic) | Unilateral leg weakness | Unilateral leg weakness or drift downwards with gravity |
|  | Reduced mobility/ Unable to mobilise | Documented inability or difficulty with standing or walking whatever the reason, including unilateral leg weakness |
|  | Unsteadiness/ ataxia | Documented to be unsteady, have poor balance or be ataxia which may be leading to reduced mobility or immobility |
|  | Visual disturbance/ changes | Any disturbance of vision including double vision / diplopia, visual field loss, loss of vision in one or both eyes. Do **not** include gaze deviation noted on examination. |
|  | Gaze deviation | Gaze deviation gaze deviation’, ‘staring/looking/gazing to left/right’ ‘eyes fixed to left/ noted on examination’ |
|  | Seizures | Include if documented seizure(s) as part of the incident. Include if described as ‘seizure’, ‘fit’, or if loss or altered consciousness with limb shaking. |
|  | Vomiting | Any vomiting as part of incident, record as no if nausea only |
|  | Difficulty swallowing | Coughing or choking on eating or drinking, or any other change in swallowing |
|  | Dizziness | Dizziness or vertigo |
|  | Leaning to one side | Noted to be leaning or slumped to one side as main complaint |
|  | Fall | Any fall from sitting or standing |
|  | Headache | Headache as part of current incident, regardless of prior history |
|  | Loss of consciousness | Complete loss of consciousness as part of current incident, regardless of whether recovered before paramedics arrive or en route to hospital |
|  | Generalised weakness | General weakness of all 4 limbs, if worse on one side mark ‘N’ and record as arm and/or leg weakness |
|  | Memory loss | Any amnesia around time of incident, either prompting the call or noted by paramedics |
|  | Behavioral changes | Any noted alteration in behavior not meeting other symptoms in list |
|  | Confusion | Clear new onset confusion, clouding of consciousness, lack of attention |
|  | Loss/ change in sensation | Loss of/ change in feeling anywhere in body as part of current incident eg numbness, paraesthesia |

**Supplementary Table S2**: Patients that died within 30 days of admission, broken down by ambulance pathway adherence, area and final diagnosis

|  | OOA suspected strokes | | | Local suspected strokes | | | All local strokes and TIAs  (n=886) |
| --- | --- | --- | --- | --- | --- | --- | --- |
|  | Stroke  (n=1697) | TIA  (n=334) | Mimic  (n=1072) | Stroke  (n=494) | TIA  (n=149) | Mimic  (n=426) |  |
| FAST- | <5 (<0.2%) | <5 (<1.5%) | <5 (<0.4%) | <5 (<1.0%) | <5  (<3.4%) | 0  (0.0%) | 7  (0.8%) |
| Pathway exclusion | 22  (1.3%) | 0 (0.0%) | 8  (0.7%) | 13  (2.6%) | 0  (0.0%) | 10  (2.3%) | 18  (2.0%) |
| Total | 204 (12.0%) | 7 (2.1%) | 63  (5.9%) | 71  (14.4%) | <5  (<3.4%) | 30  (7.0%) | 91  (10.3%) |
